# Effects of topical *Averrhoa bilimbi* leaf extract cream on IL-22 and filaggrin in a murine model of atopic dermatitis

**DOI:** 10.3389/fmed.2026.1883375

**Published:** 2026-09-25

**Authors:** Cut Putri Hazlianda, Yunita Sari Pane, Ramona Duma Sari Lubis, Andrina Yunita Murni Rambe, Khairuddin Djawad, Lidya Imelda Laksmi, Mustafa Mahmud Amin, Lia Restimulia

**Affiliations:** 1 Department of Dermatology and Venereology, Faculty of Medicine, Universitas Sumatera Utara, Medan, Indonesia; 2 Philosophy Doctor in Medicine Program, Faculty of Medicine, Universitas Sumatera Utara, Medan, Indonesia; 3 Department of Pharmacology and Therapeutics, Faculty of Medicine, Universitas Sumatera Utara, Medan, Indonesia; 4 Department of Otorhinolaryngology—Head and Neck Surgery, Faculty of Medicine, Universitas Sumatera Utara, Medan, Indonesia; 5 Department of Dermatology and Venereology, Faculty of Medicine, Hasanuddin University, Makassar, Indonesia; 6 Department of Anatomical Pathology, Faculty of Medicine, Universitas Sumatera Utara, Medan, Indonesia; 7 Department of Psychiatry, Faculty of Medicine, Universitas Sumatera Utara, Medan, Indonesia

**Keywords:** anti-inflammatory, atopic dermatitis, *Averrhoa bilimbi* Linn., filaggrin, interleukin-22

## Abstract

**Background:**

Atopic dermatitis (AD) is a chronic inflammatory skin disorder characterized by immune dysregulation and impaired epidermal barrier function. Interleukin-22 (IL-22) contributes to epidermal inflammation and disrupts keratinocyte differentiation, whereas filaggrin is essential for maintaining epidermal barrier integrity. *Averrhoa bilimbi* leaves contain phytochemical constituents with potential anti-inflammatory activity, but their effects on IL-22 and filaggrin expression in AD remain unclear.

**Objective:**

To evaluate the effects of topical *A. bilimbi* leaf ethanolic extract cream (ABLEC) on skin-tissue IL-22 concentrations and filaggrin expression in a 2,4-dinitrofluorobenzene- and ovalbumin-induced murine model of AD.

**Methods:**

This randomized posttest-only controlled animal study included 30 male BALB/c mice allocated to six groups (*n* = 5/group): vehicle control, positive control treated with 0.1% betamethasone valerate, untreated AD control, and ABLEC at 0.01%, 0.02%, and 0.04%. AD-like inflammation was induced with dinitrofluorobenzene and ovalbumin for 28 days. Assigned topical preparations were subsequently applied at 42 mg to a 6-cm^2^ dorsal skin area every 12 h for 14 days. Cutaneous IL-22 levels were measured by enzyme-linked immunosorbent assay, whereas filaggrin expression was assessed immunohistochemically and quantified using ImageJ. IL-22 data were analyzed using the Kruskal–Wallis test followed by Dunn's *post-hoc* test with Benjamini–Hochberg false-discovery-rate correction; filaggrin expression was analyzed using one-way ANOVA.

**Results:**

Cutaneous IL-22 concentrations differed significantly among groups (Kruskal–Wallis = 12.927, *p* = 0.024; *ε*^2^ = 0.330). The vehicle control, positive control, ABLEC 0.02%, and ABLEC 0.04% groups showed significantly lower IL-22 concentrations than the untreated AD control (adjusted *p* = 0.024 for each). However, none of the ABLEC groups differed significantly from the vehicle control, indicating that an extract-specific effect could not be demonstrated. Filaggrin expression did not differ significantly among groups (*p* = 0.547; *ω*^2^ = 0.000).

**Conclusion:**

ABLEC at 0.02% and 0.04% were associated with lower cutaneous IL-22 concentrations than the untreated AD control, but similar reductions in the vehicle control precluded demonstration of an extract-specific effect. Furthermore, no statistically detectable difference in filaggrin expression or dose–response relationship was observed.

## Introduction

Atopic dermatitis (AD) is a relapsing inflammatory dermatosis characterized by persistent pruritus and recurrent eczematous lesions affecting both pediatric and adult populations (1, 2). It represents a significant global health concern, with a steadily increasing prevalence (1, 3). The etiology of AD is multifactorial, resulting from intricate interactions among genetic predisposition, impaired epidermal barrier function, abnormalities of the innate immune system, and exaggerated immune responses to allergens and microbial antigens (1, 4). This pathophysiological complexity contributes to the challenges in managing AD effectively. Numerous cytokines are involved across divergent etiopathogenic pathways, and conventional treatments are largely non-specific; in severe cases, they may be insufficiently effective or be associated with significant adverse effects (2).

Existing therapeutic approaches for inflammatory skin diseases such as AD are often poorly tolerated and may lack sufficient efficacy (2, 5). Long-term use of topical corticosteroids, particularly when applied inappropriately or at unsuitable potencies, can lead to adverse effects such as cutaneous atrophy and striae. In addition, “steroid phobia” among patients, parents, and even some clinicians—driven by concerns over potential side effects—can result in poor adherence to treatment regimens (6). Consequently, traditional medicinal plants have gained attention as alternative therapeutic options (7). *Averrhoa bilimbi* Linn., commonly known as bilimbi, is widely cultivated in tropical and subtropical regions and has been traditionally employed for medicinal purposes (8, 9). Its leaves contain bioactive compounds, including saponins, tannins, flavonoids, and steroids, with reported anti-inflammatory activity. Phytochemical analysis has demonstrated total phenolic and flavonoid contents of 39.03 and 97.28 μg QE/mg, respectively. The extract also exhibited strong antioxidant activity (IC50 16.99 ± 0.12 μg/mL) and significant anti-inflammatory effects, particularly at higher concentrations (9, 10).

Interleukin-22 (IL-22) has an important role in the pathogenesis of AD, especially in preserving epithelial barrier integrity and modulating immune responses against pathogenic agents (11). Increased levels of IL-22 have been detected in the skin and serum of patients with both acute and chronic AD across different severity levels, indicating its important contribution to disease pathogenesis (12–14). IL-22 is secreted by Th22, Th17, and ILC3 cells, with Th22 cells serving as the main source in AD (11, 15). In AD, IL-22 contributes significantly to cytokine-mediated dysfunction in keratinocytes and endothelial cells (16). Increased IL-22 levels are associated with epidermal hyperplasia, acanthosis, and impaired skin barrier function (12, 14, 16, 17). Together with IL-17, IL-22 exacerbates AD by downregulating filaggrin and other genes essential for cellular adhesion, thereby worsening barrier dysfunction and amplifying inflammation (13, 16, 18, 32).

IL-22-mediated inflammation and impaired filaggrin expression represent two interrelated components of atopic dermatitis pathogenesis. Although *A. bilimbi* leaves contain several phytochemical constituents with potential anti-inflammatory activity, their effects on IL-22 and epidermal barrier protein expression have not been adequately investigated in an experimental model of atopic dermatitis. Therefore, the present study aimed to evaluate the effects of topical *A. bilimbi* leaf extract cream (ABLEC) on cutaneous IL-22 concentrations and filaggrin expression in a 2,4-dinitrofluorobenzene (DNFB)- and ovalbumin (OVA)-induced murine model of atopic dermatitis.

## Materials and methods

### Study design and animals

This *in vivo* laboratory experiment used a randomized posttest-only controlled design. Thirty healthy male BALB/c mice aged 6 weeks and weighing 20–30 g were included. Animals were excluded if they appeared ill or experienced a reduction of more than 10% in body weight during the acclimatization period. The predefined dropout criteria included death following DNFB and OVA induction, death during the experimental period, inadequate or non-evaluable skin tissue, and deterioration of the cream formulation.

### Ethical approval

All animal experimental procedures were reviewed and approved by the Health Research Ethics Committee of Universitas Sumatera Utara, Medan, Indonesia (approval no. 233/KEPK/USU/2025). The study was conducted in accordance with institutional guidelines for the care and use of laboratory animals.

### Sample-size determination

The sample size was estimated using Federer’s resource-equation approach for experimental studies:

(t−1)(n−1)>15

where *t* represents the number of experimental groups and *n* represents the number of animals in each group. With six experimental groups, the calculation yielded a minimum of four mice per group. One additional mouse was included in each group to account for potential attrition, resulting in five mice per group and a total sample size of 30 mice.

### Housing and husbandry

The mice were acclimatized to the laboratory environment for 7 days before the experimental procedures. Animals were housed in plastic cages with wood-shaving bedding and received a standard rodent diet containing 18% protein, 3.5% fat, and 5.5% fiber. Food and drinking water were provided *ad libitum*. The animal room was maintained at 22 ± 3 °C, with a relative humidity of 30%–70% and a 12-h light/12-h dark cycle. The animals were protected from discomfort, unnecessary pain, injury, disease, and excessive stress, and were provided with an adequate adaptation period. Animal care was conducted in accordance with the principles of the 3Rs and the Five Freedoms of animal welfare.

### Randomization and blinding

Following completion of the 28-day DNFB/OVA induction period, the mice were randomly allocated into six experimental groups, with five animals assigned to each group. The available experimental records did not specify the method used to generate the random allocation sequence. Clinical dermatitis severity was assessed by two dermatologists who were blinded to the group allocation.

### Animal flow

Thirty male BALB/c mice were included and, following the 28-day DNFB/OVA induction period, were randomly allocated into six experimental groups with five animals per group. Prespecified dropout criteria included death following induction, death during the experimental period, inadequate or non-evaluable skin tissue, and deterioration of the cream formulation. No deaths, exclusions, or replacements occurred during the study, and all 30 animals were included in the final analysis. The experimental animal flow is summarized in Supplementary Figure S1.

### Cutaneous sampling and euthanasia

Twenty-four hours after the final topical application, the mice were anesthetized with ketamine at 80 mg/kg body weight and xylazine at 8 mg/kg body weight administered intraperitoneally. A 6-cm^2^ dorsal skin specimen was excised symmetrically from the treatment area to minimize regional variation. The specimen was divided into two approximately equal portions: one portion was processed for enzyme-linked immunosorbent assay (ELISA) analysis, whereas the other was fixed in 10% neutral buffered formalin for histopathological and immunohistochemical examination. After completion of tissue sampling and the planned experimental assessments, the animals were euthanized by cervical dislocation and subsequently incinerated.

### Experimental groups

Following the 28-day induction period, the mice were allocated into six groups, with five mice in each group:

1. Vehicle control: AD-model mice treated with the cream base without active extract.
2. Positive control: AD-model mice treated with 0.1% betamethasone valerate cream.
3. Untreated AD control: AD-model mice that did not receive topical cream.
4. ABLEC 0.01%: AD-model mice treated with 0.01% ABLEC.
5. ABLEC 0.02%: AD-model mice treated with 0.02% ABLEC.
6. ABLEC 0.04%: AD-model mice treated with 0.04% ABLEC.

DNFB and OVA exposures were continued in all AD-model groups during the 14-day treatment period.

### Preparation of the extract

Botanical authentication of the plant material was performed by the Herbarium Medanense (MEDA), Laboratory of Plant Systematics, Universitas Sumatera Utara, Medan, Indonesia. The specimen was identified as *A. bilimbi* L. (family Oxalidaceae), and the identification was documented under certificate No. 1018/MEDA/2025, dated 14 July 2025.

*A. bilimbi* leaves were collected between 07:00 and 10:00. The leaves were washed under running water, air-dried at room temperature, and ground into a powdered simplicia. A total of 795 g of powdered leaves was macerated in 70% ethanol for three consecutive 24-h periods with intermittent stirring. The filtrate was separated and concentrated using a rotary evaporator at approximately 40 °C until a concentrated extract was obtained. The extract was stored in a tightly closed container at 4 °C until formulation.

### Phytochemical characterization

The *A. bilimbi* leaf extract was evaluated through qualitative phytochemical screening for alkaloids, flavonoids, tannins, saponins, glycosides, and terpenoid/steroid compounds. Chromatographic fingerprinting and quantitative marker-compound analyses were not performed in the present study.

### Preparation of ABLEC

ABLEC was formulated as an oil-in-water cream. The aqueous phase, consisting of purified water, triethanolamine, methyl paraben, and propyl paraben, and the oil phase, consisting of liquid paraffin and cera alba, were separately heated to 70 °C. The aqueous phase was gradually added to the oil phase with continuous stirring until an emulsion was formed. After cooling to room temperature, the extract was incorporated and mixed until a homogeneous cream was obtained.

The cream base consisted of 45% liquid paraffin, 13.5% cera alba, 2.7% triethanolamine, 0.05% methyl paraben, 0.05% propyl paraben, and purified water to 100%. The extract was incorporated at concentrations of 0.01%, 0.02%, or 0.04% w/w.

### Physical characterization of the cream

The physical properties of the ABLEC formulations were evaluated using organoleptic examination, homogeneity testing, pH measurement, viscosity measurement, spreadability testing, and cycling stability testing. Viscosity was measured using a Brookfield viscometer with spindle no. 63 at 50 rpm and 25 °C. Spreadability was evaluated by placing 0.5 g of cream between two glass plates, applying a load of 125 g, and measuring the spreading diameter.

Stability was evaluated using a cycling test in which the formulations were alternately stored at 4 °C and 40 °C for 24 h at each temperature per cycle. Changes in color, odor, consistency, homogeneity, and phase separation were recorded.

### Results of cream characterization

All three ABLEC formulations were white, had a characteristic odor, exhibited a cream-like consistency, and were homogeneous. The pH values of the ABLEC 0.01%, 0.02%, and 0.04% formulations were 6.23, 6.22, and 6.23, respectively. Their corresponding viscosity values were 555.0, 565.5, and 591.4 cP, whereas the spreadability values under a 125-g load were 6.65, 6.20, and 6.10 cm, respectively. All formulations remained physically stable during the cycling test, with no reported changes indicating formulation instability.

### Induction of atopic dermatitis-like inflammation

From days 9 to 36, AD-like inflammation was induced using 100 μL of 0.15% DNFB prepared in acetone and olive oil (3:1), applied topically to the shaved dorsal skin three times weekly, and 100 μL of 0.15% OVA prepared in normal saline, administered twice weekly as one topical application and one intraperitoneal injection. The initial 28-day induction regimen was adapted from the combined DNFB/OVA model described by Jiang et al. (19). Following the initial 28-day induction period, the mice were allocated into six experimental groups. During the subsequent 14-day treatment period, DNFB and OVA exposures were continued in all AD-model groups while the assigned topical treatments were administered.

### Treatment administration

A total of 42 mg of the assigned topical preparation was weighed using an analytical balance and applied evenly to the 6-cm^2^ dorsal treatment area. The amount of cream applied per treatment was selected based on the topical application protocol reported by Suwarsa et al. (20). The preparation was administered every 12 h for 14 consecutive days, following the treatment duration and frequency described by Frazier and Bhardwaj (21). The same amount, frequency, and duration of topical administration were applied to the vehicle-control, positive-control, and ABLEC groups to standardize topical exposure across all treatment groups. The twice-daily treatment schedule was also aligned with the administration schedule of the standard topical corticosteroid used as the positive control. The 14-day treatment period was selected as a short-term therapeutic assessment window consistent with previous experimental studies in DNFB-induced AD-like murine models in which topical interventions were administered twice daily for 14 days (22). Tissue collection 24 h after the final treatment was intended to characterize inflammatory and epidermal responses at the end of the predefined treatment period.

### Supportive clinical observation

Clinical dermatitis severity was assessed 24 h after the final topical application by two dermatologists who were blinded to the group allocation. Severity was classified as no visible rash, mild, moderate, severe, or very severe. Mild dermatitis was defined as dryness and scaling; moderate dermatitis as dryness, scaling, and erosion; severe dermatitis as dryness, scaling, erosion, and excoriation; and very severe dermatitis as dryness, scaling, erosion, excoriation, and bleeding. Clinical severity was recorded as a supportive observation and was not designated as a principal efficacy outcome.

### IL-22 measurement

Cutaneous IL-22 concentrations were quantified using a mouse IL-22 enzyme-linked immunosorbent assay kit (Elabscience®, catalog no. E-EL-M2446, Wuhan, China) according to the manufacturer's instructions. The results were expressed in pg/mL. Because of limited kit availability, each sample was measured once rather than in duplicate or triplicate.

### Filaggrin immunohistochemistry

Filaggrin expression was evaluated immunohistochemically using a specific anti-filaggrin antibody (33). Brown-positive staining was quantified using ImageJ in five microscopic fields at 200× magnification. The mean percentage of positively stained area was calculated for each animal and used in the statistical analysis.

### Statistical analysis

Data distribution was assessed using the Shapiro–Wilk test. Cutaneous IL-22 concentrations were presented as median and interquartile range and analyzed using the Kruskal–Wallis test. When the overall test was statistically significant, pairwise comparisons were performed using Dunn's test with Benjamini–Hochberg false-discovery-rate (FDR) correction across all 15 pairwise comparisons. The global effect size was expressed as epsilon-squared (*ε*^2^), whereas the pairwise effect size r was calculated as the absolute Dunn z statistic divided by the square root of the total number of observations in the two compared groups.

Filaggrin expression was presented as mean ± standard deviation. Homogeneity of variance was evaluated using Levene's test, and differences among groups were analyzed using one-way analysis of variance (ANOVA). The effect size for the ANOVA was expressed as omega-squared (*ω*^2^). All tests were two-sided, and an adjusted *p*-value <0.05 was considered statistically significant.

## Results

### Experimental animals

A total of 30 healthy male BALB/c mice, aged 6 weeks and weighing 20–30 g, were included in the study. Following acclimatization and induction of atopic dermatitis-like inflammation, the mice were allocated into six experimental groups, with five animals per group: vehicle control, positive control, untreated AD control, ABLEC 0.01%, ABLEC 0.02%, and ABLEC 0.04%. All six groups contributed five animals to the final statistical analysis. All 30 animals completed the experimental procedures, and no deaths or exclusions occurred. Five animals from each group were included in the final statistical analysis.

### Supportive clinical observation

At the final assessment, clinical dermatitis severity did not differ significantly among the six experimental groups (Kruskal–Wallis, *p* = 0.088). In the untreated AD control group, two mice (40%) had moderate dermatitis and three mice (60%) had severe dermatitis. No severe or very severe lesions were observed in the vehicle control, positive control, or ABLEC-treated groups. Because clinical severity was assessed only at the end of the experimental period, these findings were considered supportive clinical observations rather than principal efficacy outcomes. The complete distribution of clinical dermatitis severity is presented in Supplementary Table S1.

### IL-22 levels

Cutaneous IL-22 concentrations differed significantly among the six experimental groups (Kruskal–Wallis = 12.927, *p* = 0.024; *ε*^2^ = 0.330), indicating a large overall group effect (Table 1). The median IL-22 concentrations were 119.39 (IQR 97.24–122.55) pg/mL in the vehicle control, 112.01 (96.71–120.97) pg/mL in the positive control, 150.50 (148.40–152.09) pg/mL in the untreated AD control, 137.32 (100.40–138.90) pg/mL in the ABLEC 0.01% group, 112.01 (111.48–116.22) pg/mL in the ABLEC 0.02% group, and 119.39 (107.79–122.55) pg/mL in the ABLEC 0.04% group. The distribution of cutaneous IL-22 concentrations across groups is shown in Figure 1.

**Table 1:** Skin-tissue IL-22 concentrations across the experimental groups.

Group	*n*	IL-22, median (IQR), pg/mL
Vehicle control	5	119.39 (97.24–122.55)
Positive control	5	112.01 (96.71–120.97)
Untreated AD control	5	150.50 (148.40–152.09)
ABLEC 0.01%	5	137.32 (100.40–138.90)
ABLEC 0.02%	5	112.01 (111.48–116.22)
ABLEC 0.04%	5	119.39 (107.79–122.55)

Data are presented as median (interquartile range). The overall comparison was performed using the Kruskal–Wallis test. Pairwise comparisons were performed using Dunn's test with Benjamini–Hochberg false-discovery-rate correction. The distribution of cutaneous IL-22 concentrations across groups is shown in Figure 1.

**Figure 1:**
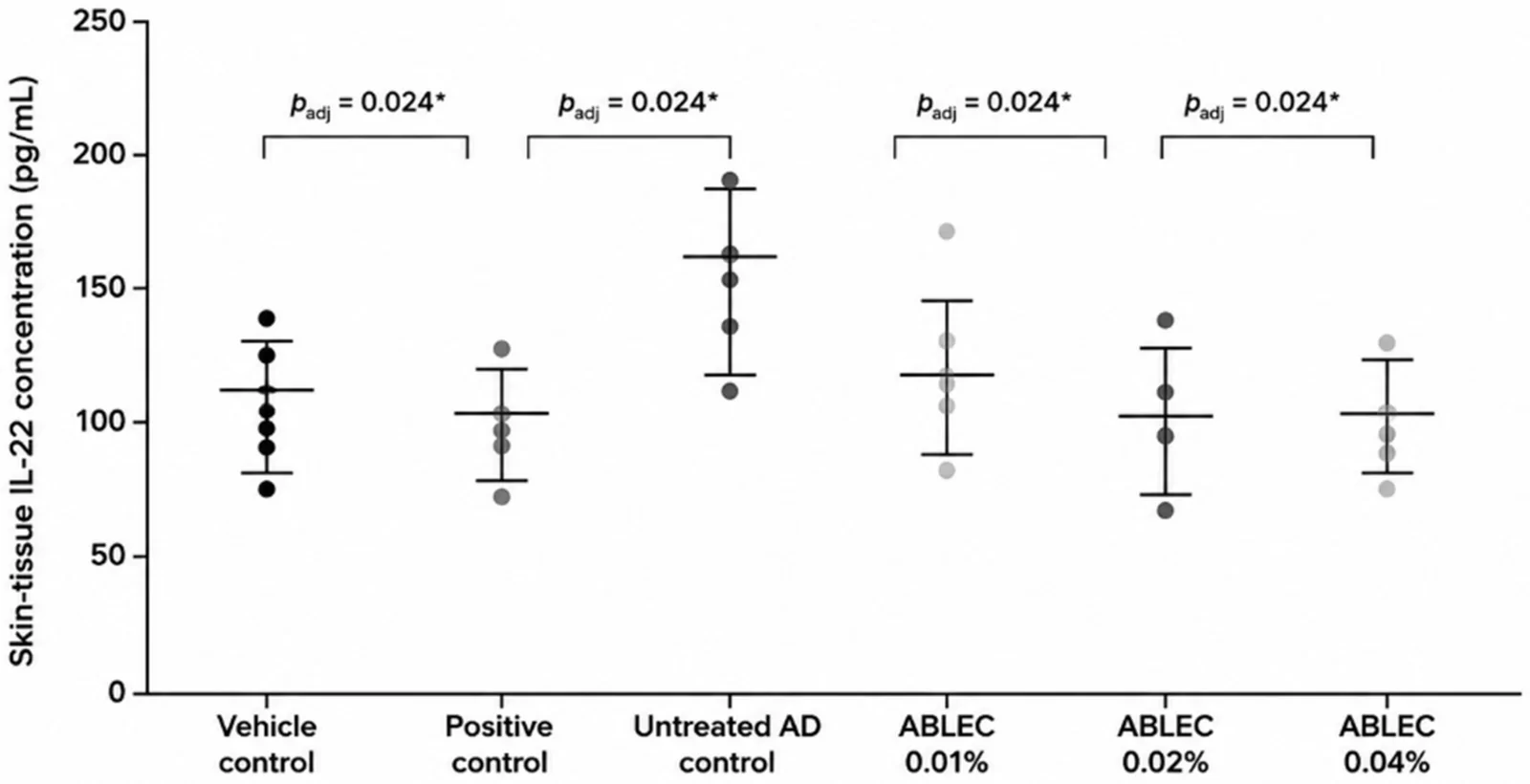
Skin-tissue IL-22 concentrations after topical administration of *A. bilimbi* leaf extract cream in a mouse model of atopic dermatitis.

After correction for multiple comparisons, the vehicle control, positive control, ABLEC 0.02%, and ABLEC 0.04% groups showed significantly lower IL-22 concentrations than the untreated AD control (adjusted *p* = 0.024 for each comparison; Table 2). Although the unadjusted comparison between ABLEC 0.01% and the untreated AD control yielded *p* = 0.048, this difference was no longer statistically significant after false-discovery-rate correction (adjusted *p* = 0.144).

**Table 2:** *Post-hoc* Dunn test with Benjamini–Hochberg FDR correction for IL-22 levels.

Comparison	Unadjusted *p*	FDR-adjusted *p*	Effect size r
Vehicle vs. positive control	0.843	1.000	0.062
Vehicle vs. untreated AD control	0.006	**0** **.** **024**	0.864
Vehicle vs. ABLEC 0.01%	0.450	0.751	0.239
Vehicle vs. ABLEC 0.02%	0.872	1.000	0.051
Vehicle vs. ABLEC 0.04%	1.000	1.000	0.000
Positive vs. untreated AD control	0.003	**0**.**024**	0.926
Positive vs. ABLEC 0.01%	0.341	0.751	0.301
Positive vs. ABLEC 0.02%	0.971	1.000	0.011
Positive vs. ABLEC 0.04%	0.843	1.000	0.062
Untreated AD control vs. ABLEC 0.01%	0.048	0.144	0.625
Untreated AD control vs. ABLEC 0.02%	0.004	**0**.**024**	0.915
Untreated AD control vs. ABLEC 0.04%	0.006	**0**.**024**	0.864
ABLEC 0.01% vs. ABLEC 0.02%	0.360	0.751	0.290
ABLEC 0.01% vs. ABLEC 0.04%	0.450	0.751	0.239
ABLEC 0.02% vs. ABLEC 0.04%	0.872	1.000	0.051

Dunn’s test with FDR correction using the Benjamini–Hochberg method. Interpretation: r < 0.10 (negligible), 0.10–0.29 (small), 0.30–0.49 (moderate), ≥0.50 (large).

Bold values indicate statistically

significant FDR-adjusted *p*-values (*p* < 0.05).

No significant differences were observed among the three ABLEC concentrations (adjusted *p* ≥ 0.751). Furthermore, none of the ABLEC groups differed significantly from the vehicle control or positive control. Therefore, the findings did not demonstrate either an extract-specific effect beyond that of the cream vehicle or a dose–response relationship within the tested concentration range.

### Filaggrin expression

Filaggrin expression did not differ significantly among the six experimental groups (one-way ANOVA = 0.821, *p* = 0.547; *ω*^2^ = 0.000) (Table 3). Mean filaggrin expression was 4.22% ± 4.79% in the vehicle control, 3.12% ± 1.09% in the positive control, 1.53% ± 0.47% in the untreated AD control, 3.32% ± 2.17% in the ABLEC 0.01% group, 3.51% ± 1.93% in the ABLEC 0.02% group, and 2.10% ± 1.62% in the ABLEC 0.04% group. These results provide no statistical evidence that ABLEC altered filaggrin expression under the experimental conditions used.

**Table 3:** Filaggrin expression across the experimental groups.

Group	*n*	Filaggrin expression, mean ± SD, %
Vehicle control	5	4.22 ± 4.79
Positive control	5	3.12 ± 1.09
Untreated AD control	5	1.53 ± 0.47
ABLEC 0.01%	5	3.32 ± 2.17
ABLEC 0.02%	5	3.51 ± 1.93
ABLEC 0.04%	5	2.10 ± 1.62

Data are presented as mean ± standard deviation. The overall comparison was performed using one-way analysis of variance. The estimated effect size was *ω*^2^ = 0.000.

## Discussion

The epidermal barrier is maintained by terminally differentiated keratinocytes and structural proteins, including filaggrin, which contribute to epidermal integrity and the limitation of transepidermal water loss (23, 24). In AD, barrier dysfunction results from complex interactions between intrinsic epidermal abnormalities and cytokine-mediated impairment of keratinocyte differentiation (23, 24). IL-22, produced predominantly by Th22 and Th17 cells, is increased in both acute and chronic AD and contributes to epidermal hyperplasia, impaired keratinocyte differentiation, and disruption of barrier function (24, 25). Increased IL-22 activity has also been associated with altered epidermal homeostasis and deterioration of skin barrier integrity (25). The Th17/IL-22 axis may be particularly relevant in chronic AD and in specific disease phenotypes, including pediatric and Asian populations (24).

In the present study, cutaneous IL-22 concentrations differed significantly among the experimental groups. After correction for multiple comparisons, the ABLEC 0.02% and ABLEC 0.04% groups showed significantly lower IL-22 concentrations than the untreated AD control, whereas the difference between ABLEC 0.01% and the untreated AD control did not remain statistically significant after false-discovery-rate correction. Importantly, the vehicle control also showed significantly lower IL-22 concentrations than the untreated AD control, and none of the ABLEC groups differed significantly from the vehicle control. Therefore, although ABLEC at 0.02% and 0.04% was associated with lower IL-22 concentrations relative to untreated AD-model mice, the present data do not demonstrate that this reduction was attributable specifically to *A. bilimbi* leaf extract.

The effect observed in the vehicle-control group warrants particular consideration. The cream base contained 45% liquid paraffin and 13.5% cera alba, and the high proportion of liquid paraffin may have provided an occlusive and emollient effect independently of the plant extract. *In vivo* studies have demonstrated that paraffin oil remains predominantly within the superficial layers of the stratum corneum and reduces transepidermal water loss through semi-occlusion of the skin surface (26). Studies of other hydrocarbon-based occlusive moisturizers, particularly petrolatum, have also demonstrated effects extending beyond simple physical occlusion, including modulation of epidermal differentiation and cutaneous barrier-related immune responses (27). Thus, improvement of the local barrier microenvironment by the cream vehicle provides a biologically plausible explanation for the lower IL-22 concentrations observed in the vehicle-control group. However, because transepidermal water loss, skin hydration, and vehicle-component-specific effects were not measured, the mechanism and magnitude of the vehicle contribution cannot be determined from the present study.

No significant differences were observed among the three ABLEC concentrations, and the present findings therefore do not demonstrate a dose–response relationship within the tested concentration range. Although previous studies have reported that certain herbal formulations may suppress inflammatory responses associated with AD and modulate JAK/STAT signaling, these pathways were not directly evaluated in the present study (24, 28). Accordingly, the involvement of JAK/STAT signaling in the observed changes in IL-22 cannot be inferred from the current data.

Several non-mutually exclusive explanations may account for the absence of a monotonic dose–response relationship across the tested ABLEC concentrations. First, the biological response may have approached a plateau within the relatively narrow concentration range of 0.01%–0.04%, such that increasing the extract concentration did not produce a proportionally greater change in cutaneous IL-22. Second, a higher concentration of extract within a conventional cream formulation may not necessarily result in proportionally greater cutaneous exposure if release from the vehicle or penetration through the stratum corneum becomes rate-limiting. Third, *A. bilimbi* leaf extract represents a complex mixture of phytochemical constituents, and interactions among individual constituents could potentially result in a non-linear concentration–response relationship. However, cutaneous deposition, penetration kinetics, and quantitative concentrations of individual phytochemical constituents were not assessed. These explanations should therefore be regarded as hypotheses for future investigation rather than mechanisms established by the present study.

In contrast to the findings for IL-22, filaggrin expression did not differ significantly among the experimental groups. Although mean filaggrin expression was numerically higher in some ABLEC-treated groups than in the untreated AD control, these descriptive differences should not be interpreted as evidence of improved epidermal barrier protein expression. Given the small number of animals per group and the use of Federer's resource-equation approach rather than an *a priori* power calculation based on a prespecified effect size, the present finding is more appropriately interpreted as an absence of a statistically detectable between-group difference under the experimental conditions used, rather than definitive evidence that ABLEC has no biological effect on filaggrin expression.

Previous studies have suggested that plant-derived compounds may influence filaggrin expression through several pathways, including activation of the aryl hydrocarbon receptor (AhR), modulation of STAT6 signaling, and activation of downstream transcriptional regulators such as OVOL1 (29, 30, 34). However, AhR, STAT6, OVOL1, and other molecular regulators of epidermal differentiation were not evaluated in the present study. Consequently, these pathways represent possible mechanisms described in the literature but cannot be attributed to *A. bilimbi* leaf extract on the basis of the present findings.

Kim et al. reported that *Pulsatilla koreana* Nakai extract increased the expression of filaggrin, involucrin, and loricrin at higher concentrations. Molecular docking analysis further suggested that several constituents of the extract, including chicoric acid, anemoside B4, hederacoside C, hederacoside D, hederasaponin B, anemoside A3, and *α*-hederin, may interact with components of the JAK/STAT signaling pathway (31). However, those findings were obtained using a different botanical extract, formulation, experimental model, and molecular approach. They therefore provide biological context for potential plant-derived modulation of epidermal barrier pathways but do not constitute evidence that *A. bilimbi* leaf extract acts through the same mechanisms.

The dissociation between lower cutaneous IL-22 concentrations and the absence of a statistically detectable change in filaggrin expression may reflect differences in the kinetics of inflammatory and structural responses in AD. Changes in inflammatory mediators may occur before the measurable restoration of epidermal structural proteins. Alternatively, the absence of a detectable filaggrin response may have been influenced by the 14-day treatment period, limited statistical power, variability in immunohistochemical measurements, or limited cutaneous delivery of active constituents from the conventional cream formulation. These factors provide biologically plausible explanations for the observed dissociation; however, none was directly evaluated in the present study. Accordingly, causal inferences regarding treatment duration, cutaneous penetration, or the molecular regulation of filaggrin cannot be made from the current data. Future studies incorporating serial measurements during and after treatment would help determine whether modulation of inflammatory mediators precedes, coincides with, or occurs independently of changes in epidermal structural proteins.

Another important consideration is the limited chemical standardization of the extract. The extract was characterized using qualitative phytochemical screening, whereas chromatographic fingerprinting and quantitative marker-compound analyses were not performed. Consequently, the observed biological activity cannot be linked to defined concentrations of specific constituents, and chemical comparability between different extract batches cannot be established. This issue is particularly relevant for botanical preparations because the reproducibility of biological effects depends on the adequate characterization and standardization of the tested material. Future confirmatory studies should therefore prioritize chromatographic fingerprinting using methods such as high-performance liquid chromatography (HPLC) or high-performance thin-layer chromatography (HPTLC), quantitative determination of selected marker compounds, and predefined batch-to-batch specifications before further efficacy testing. Such characterization would allow subsequent biological responses to be interpreted in relation to a reproducible phytochemical profile.

In addition, the significant reduction in cutaneous IL-22 observed in the vehicle-control group indicates that the biological contribution of the cream base must be distinguished from that of the plant extract. Accordingly, chemical standardization of the extract, including chromatographic fingerprinting and quantitative marker-compound analysis, together with dedicated vehicle-dissection experiments designed to distinguish vehicle-mediated from extract-specific effects, is a prerequisite before ABLEC-specific bioactivity can be established.

This study has several limitations. First, the sample size was determined using Federer's resource-equation approach rather than an *a priori* power calculation based on a prespecified effect size. Therefore, the study may have had limited statistical power to detect small or moderate between-group differences, particularly in filaggrin expression. Second, clinical dermatitis severity was assessed only at the end of the experimental period; therefore, baseline comparability immediately before treatment allocation and within-animal changes during treatment could not be evaluated. Third, outcomes were evaluated at a single posttreatment time point, limiting assessment of the temporal relationship between changes in inflammatory mediators and epidermal structural proteins. Fourth, transepidermal water loss, skin hydration, scratching behavior, epidermal thickness, and inflammatory-cell infiltration were not assessed, limiting interpretation of functional and histological barrier changes and of the potential contribution of the cream vehicle. Fifth, each ELISA sample was measured once rather than in duplicate or triplicate, limiting the assessment of analytical reproducibility. Sixth, chromatographic fingerprinting and quantitative marker-compound analyses were not performed, limiting chemical standardization and reproducibility of the botanical preparation. Seventh, formal longitudinal body-weight monitoring, standardized skin-irritation assessment, and comprehensive toxicity evaluation were unavailable. Eighth, the stratification factor and method used to generate the allocation sequence were not sufficiently documented. Finally, molecular pathways including JAK/STAT and AhR/OVOL1 were not directly evaluated; therefore, the involvement of these pathways cannot be inferred from the present findings.

## Conclusion

ABLEC at concentrations of 0.02% and 0.04% was associated with lower cutaneous IL-22 concentrations compared with untreated AD-model mice, whereas the difference observed with ABLEC 0.01% did not remain significant after correction for multiple comparisons. However, none of the ABLEC groups differed significantly from the vehicle control, indicating that an extract-specific reduction in IL-22 was not demonstrated. No statistically detectable difference in filaggrin expression was observed among the experimental groups, and no dose–response relationship was identified across the tested ABLEC concentrations.

## Data Availability

The original contributions presented in the study are included in the article/Supplementary Material, further inquiries can be directed to the corresponding author.
