# Supplementary material for: Effects of topical Averrhoa bilimbi leaf extract cream on IL-22 and filaggrin in a murine model of atopic dermatitis
Source: Front Med (Lausanne). 2026 Sep 25;13:1883375. doi: 10.3389/fmed.2026.1883375 (PMC13649757; doi:10.3389/fmed.2026.1883375)
Supplement: Supplementary file 1 [file Supplementaryfile1.docx]

**Supplementary File**

Supplementary Table S1. Clinical dermatitis severity at the end of the experimental period

| **Group** | **No visible rash** | **Mild** | **Moderate** | **Severe** | **Very severe** | **p** |
| --- | --- | --- | --- | --- | --- | --- |
| Vehicle control | 2 (40) | 2 (40) | 1 (20) | 0 | 0 | 0.088* |
| Positive control | 3 (60) | 1 (20) | 1 (20) | 0 | 0 |  |
| Untreated AD control | 0 | 0 | 2 (40) | 3 (60) | 0 |  |
| ABLEC 0.01% | 3 (60) | 1 (20) | 1 (20) | 0 | 0 |  |
| ABLEC 0.02% | 2 (40) | 1 (20) | 2 (40) | 0 | 0 |  |
| ABLEC 0.04% | 3 (60) | 0 | 0 | 2 (40) | 0 |  |

Data are presented as n (%). Overall comparison was performed using the Kruskal–Wallis test. Clinical dermatitis severity was recorded as a supportive observation and was not designated as a principal efficacy outcome.

**Supplementary Figure S1. Animal flow diagram of the experimental study.** Thirty male BALB/c mice underwent a 7-day acclimatization period followed by 28 days of DNFB/OVA induction. The mice were subsequently randomly allocated into six experimental groups (n = 5/group): vehicle control, positive control, untreated AD control, and ABLEC 0.01%, 0.02%, and 0.04%. After the 14-day treatment period, clinical assessment and skin-tissue sampling were performed, followed by euthanasia and laboratory analyses of IL-22 and filaggrin.
